# Development of a liposomal nanodelivery system for nevirapine

**DOI:** 10.1186/1423-0127-17-57

**Published:** 2010-07-13

**Authors:** Lakshmi N Ramana, Swaminathan Sethuraman, Udaykumar Ranga, Uma M Krishnan

**Affiliations:** 1 Centre for Nanotechnology & Advanced Biomaterials (CeNTAB), School of Chemical & Biotechnology, SASTRA University, Thanjavur 613 401, India; 2 Molecular Virology Laboratory, Molecular Biology & Genetics Unit, Jawaharlal Nehru Centre for Advanced Scientific Research, Bangalore 560 064, India

## Abstract

**Background:**

The treatment of AIDS remains a serious challenge owing to high genetic variation of Human Immunodeficiency Virus type 1 (HIV-1). The use of different antiretroviral drugs (ARV) is significantly limited by severe side-effects that further compromise the quality of life of the AIDS patient. In the present study, we have evaluated a liposome system for the delivery of nevirapine, a hydrophobic non-nucleoside reverse transcriptase inhibitor. Liposomes were prepared from egg phospholipids using thin film hydration. The parameters of the process were optimized to obtain spherical liposomes below 200 nm with a narrow polydispersity. The encapsulation efficiency of the liposomes was optimized at different ratios of egg phospholipid to cholesterol as well as drug to total lipid. The data demonstrate that encapsulation efficiency of 78.14% and 76.25% were obtained at egg phospholipid to cholesterol ratio of 9:1 and drug to lipid ratio of 1:5, respectively. We further observed that the size of the liposomes and the encapsulation efficiency of the drug increased concomitantly with the increasing ratio of drug and lipid and that maximum stability was observed at the physiological pH. Thermal analysis of the drug encapsulated liposomes indicated the formation of a homogenous drug-lipid system. The magnitude of drug release from the liposomes was examined under different experimental conditions including in phosphate buffered saline (PBS), Dulbecco's Modified Eagle's Medium (DMEM) supplemented with 10% fetal bovine serum or in the presence of an external stimulus such as low frequency ultrasound. Within the first 20 minutes 40, 60 and 100% of the drug was released when placed in PBS, DMEM or when ultrasound was applied, respectively. We propose that nevirapine-loaded liposomal formulations reported here could improve targeted delivery of the anti-retroviral drugs to select compartments and cells and alleviate systemic toxic side effects as a consequence.

## Introduction

According to the World Health Organization, more than 40 million people have been presently infected with Human Immunodeficiency Virus type 1 (HIV-1) globally. Highly Active Antiretroviral Therapy (HAART), which consists of a combination of a minimum of three antiretroviral (ARV) drugs, is the primary treatment currently available for efficient management of AIDS [1,2]. The various types of ARVs that are used in HAART could be categorized into nucleoside reverse transcriptase inhibitors (NRTIs), nucleotide reverse transcriptase inhibitors (nRTIs), non-nucleoside reverse transcriptase inhibitors (NNRTIs), protease inhibitors (PIs), viral fusion inhibitors, integrase inhibitors, maturation inhibitors and fixed dose combination [1]. These drugs have a potential to manage the chronic infection but not to treat the disease [3]. The bioavailability of many of the ARV drugs is considerably low and erratic due to the substantial first pass metabolism and degradation in the gastrointestinal tract. Given the short half-life of the drugs, frequent administration of the drugs is required at relatively higher doses, often leading to low patient compliance [4]. If adherence falls below 95% level, the therapeutic effectiveness is reduced below 50% [3]. Immunologically privileged compartments of the body including the central nervous system, lymphatic system and the macrophages are characteristically inaccessible to a majority of the ARV drugs thus serving as viral reservoirs [5]. The inability to maintain therapeutic concentration of the drugs for longer durations significantly contributes to multidrug-resistance [6]. Furthermore, the prolonged use of ARVs frequently leads to toxic side effects resulting in the deterioration in the quality of life and incompliance to therapy [7]. Nevirapine is a hydrophobic NNRTI that non-competitively binds to an allosteric non-substrate binding site of the reverse transcriptase (RT) [8,9]. Nevirapine, the first ARV member of non-nucleoside reverse transcriptase inhibitor approved by the Food and Drug Administration (FDA) for HIV and an important component of HAART, is typically the primary choice for efficient viral suppression. Unfortunately, the use of nevirapine is frequently accompanied by severe side-effects that include CNS toxicity, hepatotoxicity, insomnia, confusion, memory loss, depression, rash, nausea, dizziness, Stevens-Johnson syndrome, toxic epidermal necrolysis and hyperlipidemia [8-16]. It has also been reported to cause severe liver toxicity within first six weeks of treatment. The US FDA has issued a 'black box label' on nevirapine due to its hepatotoxicity [14]. The use of nevirapine has been restricted except in cases where the benefit to the patient exceeds the risk. Therefore the development of a delivery system for sustained and targeted release of nevirapine can enhance the clinical potential of this antiretroviral drug. Nevirapine also reduces the level of certain co-administered drugs including the antiretroviral drugs indinavir, lopinavir, efavirenz. In order to prevent such undesired interactions, encapsulation of nevirapine in a carrier is expected to be beneficial. Given the paradoxical context, there exists a need to develop targeted and sustained drug delivery systems to reduce the frequency of dose administration on the one hand and to maintain therapeutic concentration of the drug for extended periods with enhanced efficacy on the other hand which could also improve patient compliance. The liposomal carrier system is expected to reduce the side-effects due to sustained release of the drug and provide sufficient cellular uptake due to its nano-dimensions.

A range of novel strategies are currently being developed for efficient delivery of ARV drugs. Efficient delivery could be achieved by encapsulating the drug or by attaching it with a carrier system [17-19]. Several delivery systems have been reported for the delivery of ARV drugs including bioadhesive coated matrix tablets [20,21], ceramic implants [22], liposomes [23-26], solid colloidal nanoparticles [27-30], dendrimers [31], micelles & microemulsion [32], nanopowders [33] and suspensions [34]. Liposomes are nanocarriers that range from 25 nm to several microns and are prepared using combinations of natural or synthetic phospholipids and cholesterol [35]. Liposomes incorporate hydrophilic drugs through an aqueous core or entrap hydrophobic drugs using phospholipid bilayer(s) which surrounds the aqueous core. Since some of the cells of the immune system like the macrophages and microglial cells could serve as the viral reservoirs, liposomes could potentially target ARV drugs into the infected cells thereby improving the efficacy and reducing the side-effects [36]. The primary aim of the present study was to develop and characterize nevirapine-loaded liposomes and to investigate the effect of various parameters on the size and the encapsulation efficiency of the liposomes including the lipid composition, drug-lipid ratio and pH of the medium. The release kinetics of nevirapine in solutions at varying pH and culture medium in the presence and absence of an external stimulus were determined.

## Materials and methods

### Materials

Methanol, phosphotungstic acid, sodium chloride, sodium dihydrogen phosphate, disodium hydrogen phosphate, sucrose, chloroform, hydrochloric acid were purchased from Merck Chemicals, India and used as such without further purification. Egg phosphatidyl choline (EPC) was procured from Sigma-Aldrich, USA. Nevirapine was a kind gift from Bohringer Ingelheim, Germany.

### Preparation of Liposomes

Egg phospholipids were extracted from yellow yolk by the modified Singleton-Gray method [37]. The lipid composition of egg phospholipids have been identified using the GC-MS (Agilent technologies, Model 7890 A series, GC with 5975C Mass spectrometer). The results indicate that the egg phospholipid contains three different lipid constituents such as PLPC (89%), POPE (3%) and cholesterol (6%). Liposomes were prepared using the thin film hydration technique. Briefly, 100 mg/mL of phospholipids in chloroform taken in a clean moisture-free container was purged with nitrogen gas to remove the solvent. Five mL of phosphate buffered saline (PBS), pH 7.4, were added to the container and the mixture was warmed at 60°C for 30 minutes. The solution was then extruded through polycarbonate membranes of 200 nm pore size using an extruder (Liposofast Basic, Avestin, Canada) for ten cycles to obtain extruded liposomes. The liposomes were lyophilized (Virtis Model Benchtop K, USA) and stored at -20 °C in air-tight vials.

### Drug Loading

Nevirapine loaded liposomes were prepared dissolving eight different ratios of drug to phospholipids (1:1, 1:2, 1:3, 1:4, 1:5, 1:6, 1:7 and 1:10). Briefly, a total amount of lipid consisting of 10 mg of phospholipids in chloroform and different quantities of nevirapine was dissolved in chloroform and the liposomes were prepared as explained above.

### Morphological Characterization

The morphology of the liposomes was determined using a scanning electron microscope (JEOL 6701F, Japan). The samples were placed over a carbon paste coated stub and sputter coated with a thin layer of platinum prior to viewing. For negative staining, 2% (w/v) phosphotungstic acid was added to the liposome samples and incubated at room temperature for 24 hours. This sample was freeze dried and imaged using scanning electron microscope. The transmission electron micrographs of the liposomes were obtained using JEM 1011, JEOL, Japan. The lyophilized liposome sample was dispersed in 0.5 mL PBS. To 50 μL of this dispersion, an equal volume of double distilled water was added and placed on a carbon coated grid. The excess water was absorbed using a filter paper and uranyl acetate stain was added. The grid was then washed with water to remove excess uranyl acetate and then dried before it was loaded in the specimen chamber. The percentage aqueous volume of liposome was calculated using the formula

### Particle Size Analysis

The particle size of the liposomes and drug loaded liposomes were determined using laser diffraction method (Microtrac Blue wave, Japan) at room temperature. Five mL of the sample was introduced into the particle size analyzer at 50% flow rate to measure the mean size and size distribution of liposomes and drug loaded liposomes.

### Thermal Analysis

Two mg of liposome samples were loaded in aluminum pans along with the standard reference aluminum in the differential scanning calorimeter (Q20, TA Instruments, USA). The DSC was recorded between 10°C and 90°C at a scan rate of 10°C/min for three cycles.

### Determination of Encapsulation Efficiency

The extruded liposomal samples were centrifuged at 3,000 rpm (Eppendorf 3340R, Germany) at 4°C to pelletize the unencapsulated drug. The supernatant was centrifuged at 10,000 rpm to pelletize the drug loaded liposomes [38]. The pellet was then treated with 1% Triton X-100 (Sigma-Aldrich, USA) to disrupt the liposomes. The sample was centrifuged at 3000 rpm again to pelletize the drug alone. The supernatant was removed and the pellet was resuspended and the concentration of the encapsulated drug was measured as absorbance at 284 nm using UV-visible spectrophotometer (Lambda 25, Perkin Elmer, USA). The absorbance was converted into drug concentration using a standard curve.

The encapsulation efficiency was calculated as:

All experiments were carried out in triplicate.

### Release Kinetics

Dialysis bags (Dialysis membrane 110, Hi Media, India) were immersed in water for one hour to remove the preservatives followed by rinsing in phosphate buffered saline (PBS) solution. The drug encapsulated liposomes were placed in PBS and loaded in the dialysis bag. The bag was sealed at both the ends and immersed in 4 mL of PBS with 10% methanol [39]. The release of the drug was evaluated at three different pH values (1.2, 7.4 and 9.0). A pH of 1.2 was maintained using 0.1 M HCl -KCl buffer while pH 9.0 was maintained using 0.1 M phosphate buffer. In order to evaluate the influence of proteins on the release of nevirapine from the liposomes, Dulbecco's Modified Eagle's Medium (DMEM, HiMedia, India) supplemented with 10% fetal bovine serum (FBS, HiMedia, India) was used as the release medium. The effect of ultrasound on the release profile of the drug from the liposomes was studied in PBS (pH 7.4) as the release medium. Low frequency ultrasound (20 KHz) was applied using a bath sonicator (UT 002, ABM, India) for the entire duration of the release study. For all drug release studies, 4 ml of the release medium was withdrawn for analysis at different time intervals (0-25 hours) and replaced with 4 mL of fresh medium. The amount of drug released was measured as absorbance using a UV-visible spectrophotometer (Lambda 25, Perkin Elmer, USA). The absorbance was converted into percentage release using a standard curve.

### Statistics

Analysis of Variance (Two-way Anova) was performed to determine the statistical significance (p < 0.05) for percentage encapsulation (n = 3) and percentage of drug release (n = 3) under various experimental conditions. If statistically significant, a post-hoc Tukey test was performed to determine which means were different from the others.

## Results & Discussion

**Figure 1:**
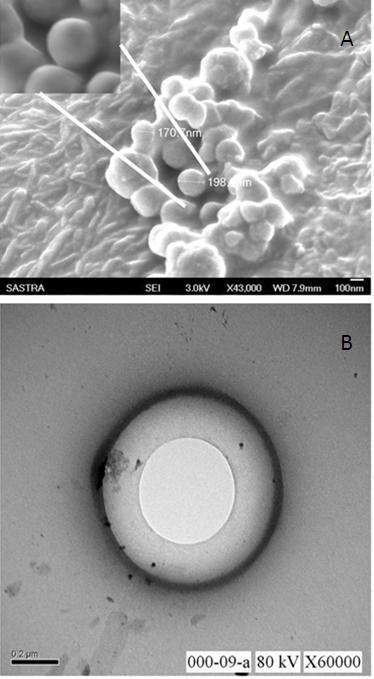
**SEM and TEM micrographs of the lyophilized liposomes**. (A) The sample was imaged s performed at a magnification of 43,000. Inset shows the magnified image of spherical liposome (B) TEM micrographs of liposome at a magnification of 60,000.

The mean particle size of the liposomes prepared using thin film hydration technique was 157 nm. The scanning electron micrograph of the lyophilized liposome indicates a spherical morphology and size in the nanodimensions (Figure 1A). Figure 1B presents the transmission electron micrograph of the liposomes clearly demarcating an aqueous phase in the centre of the liposome. The average aqueous volume of the liposomes determined from the various transmission electron micrographs is 15.54%. This small aqueous volume is likely due to the small vesicle sizes obtained in which considerable volume is occupied by the membrane [40].

**Figure 2:**
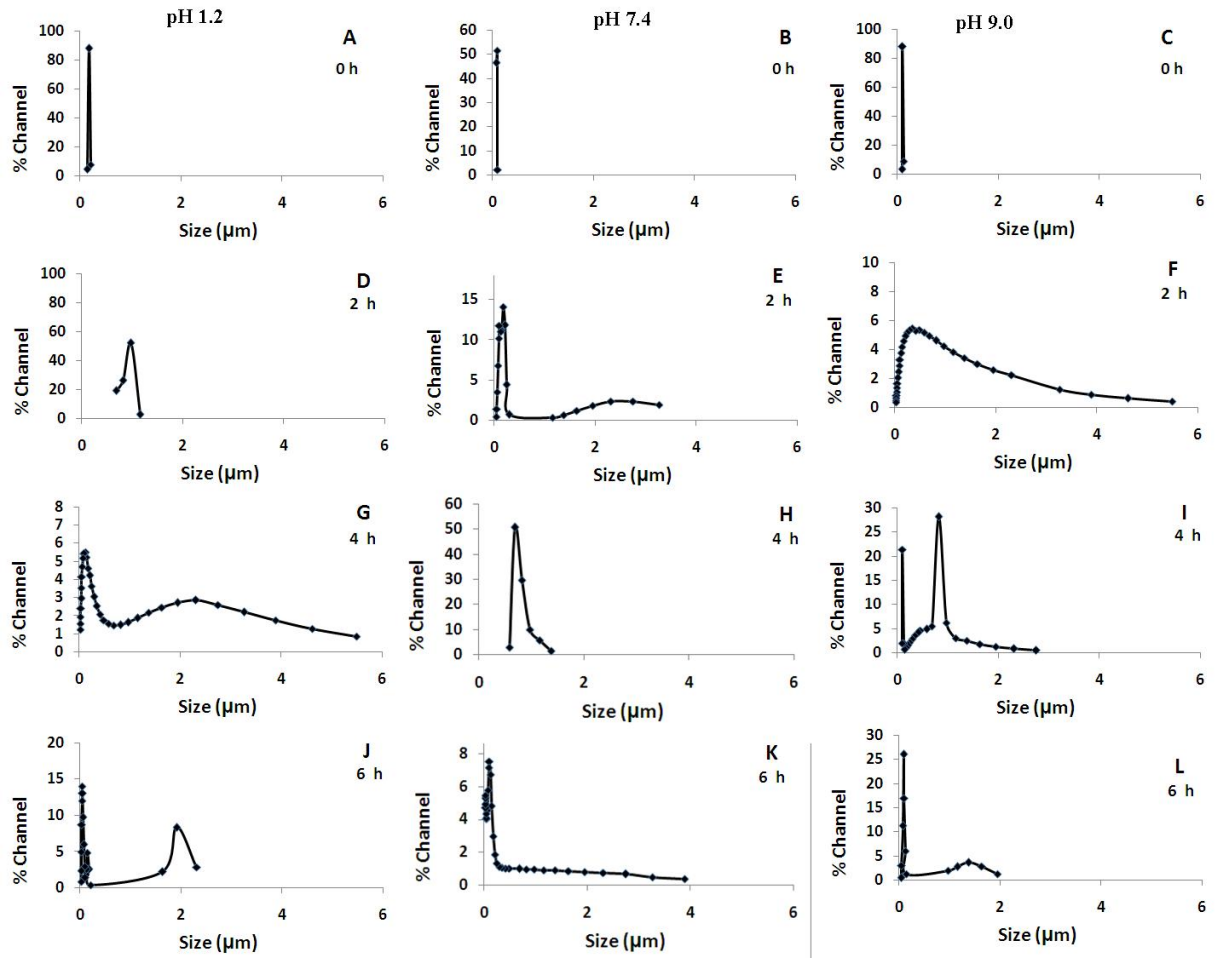
**Effect of pH on colloidal stability of liposome**.

The colloidal stability of the liposomes was investigated at three different pH conditions, 1.2, 7.4 and 9.0 at the end of incubation for defined time points (Figure 2). We observed that the size of the liposomes was significantly influenced by the pH of the medium. The size of the liposomes changed significantly at both acidic (1.2) and basic pH (9.0) when compared to the neutral pH (7.4). For the first two hours, the liposome size increased regardless of the pH differences (Figure 2, panels D, E & F). This may be attributed to the secondary particle growth due to vesicle fusion or Ostwald ripening which is expected to be promoted by small vesicles because of their high membrane curvature [41,42]. At later time points, the size of the liposomes at acidic and alkaline pH showed a significant decrease as compared to pH 7.4 (Figure 2, panels G, H & I). This may be attributed to the competitive hydrolysis of the phospholipids that may occur spontaneously in the media resulting in the destruction of the liposomal architecture leading to size reduction. Further, increased accumulation of dipoles at the membrane interface may also lead to reduced aggregation [43]. However, no such size reduction was observed at pH 7.4 instead a gradual increase in the liposomal size towards the micron range was noticed due to a major contribution from Ostwald ripening (Figure 2). With increasing salt concentration the liposomes start to aggregate. However, as hydrolysis starts dominating, the charged phosphate head groups are hydrolyzed leading to a reduction of charge and hence an associated reduction in the micron sized liposomes [44].

After the initial aggregation, two distinct size groups of liposomes - nano and micro size liposomes were formed at both acidic (Figure 2G) and alkaline pH conditions (Figure 2I). As a function of time the size of the particles decreased and larger fraction of the liposomes were seen in the nanoscale range at both acidic (Figure 2J) and basic (Figure 2L) pH conditions.

**Figure 3:**
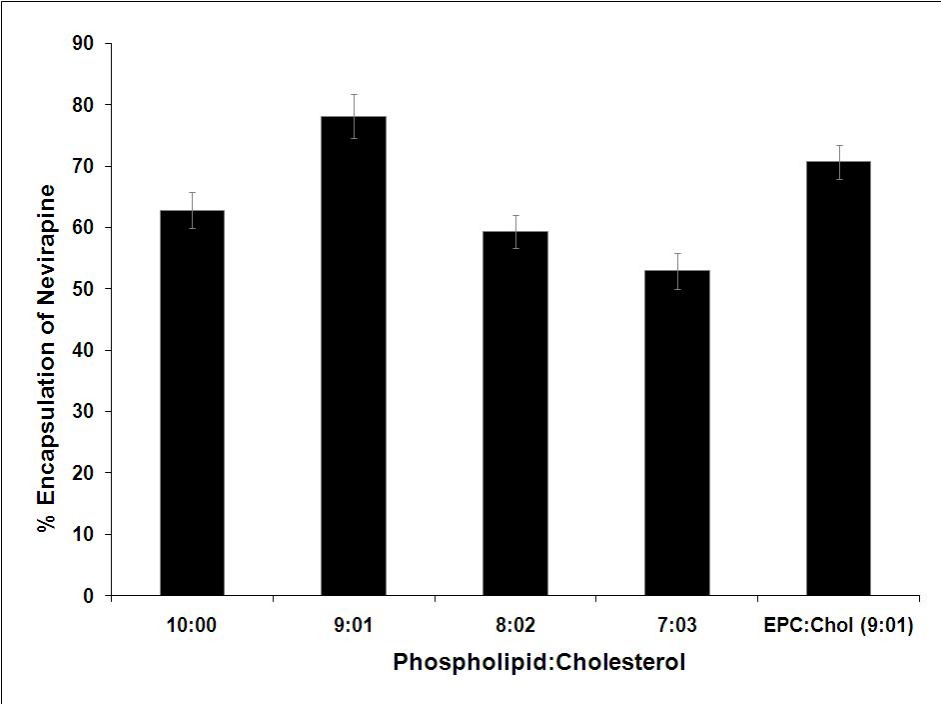
**Effect of Cholesterol on the encapsulation efficiency of nevirapine. Statistical data infers that each group is significantly different (p < 0.05)**.

**Figure 4:**
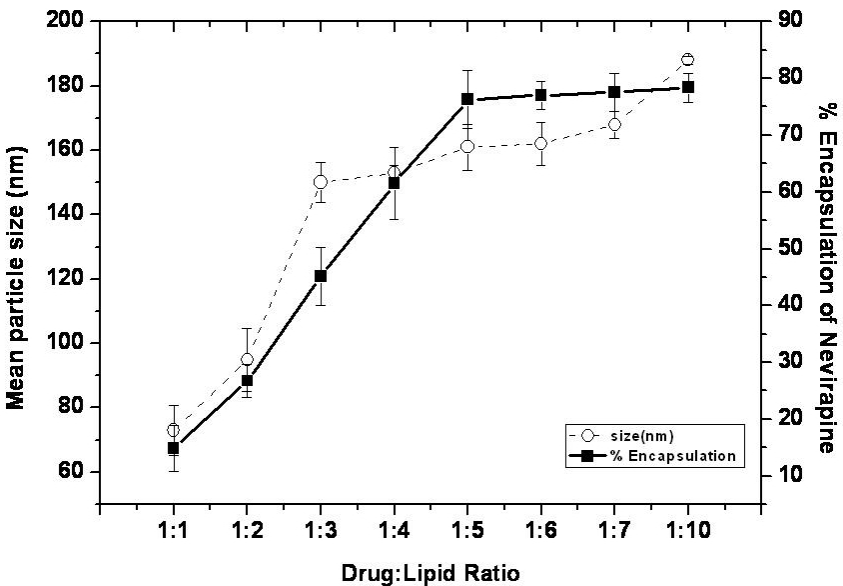
**Correlation between the mean particle size (white circle) and percent encapsulation efficiency (Black square) at various drug-lipid ratios**.

Since lipid composition could have significant impact on liposome size, stability, drug loading and delivery functions, we examined the physical properties of the liposomes synthesized at varying ratios of egg phospholipids and cholesterol. The encapsulation efficiency of liposomes constituted from varying concentrations of phospholipid and cholesterol for nevirapine loading was compared (Figure 3). The encapsulation efficiency of the liposomes was significantly influenced by the presence of cholesterol and its drug to lipid ratio. Liposomes consisting of cholesterol (at 9:1 ratio) showed significantly increased encapsulation efficiency for nevirapine as compared to the particles without cholesterol (at 10:0 ratio, p < 0.05). The enhanced loading capacity of the liposomes may be attributed to a combined effect of increased hydrophobicity and or increased liposome size due to cholesterol incorporation [45]. Importantly, further increase in the cholesterol content did not enhance drug loading capacity of the liposomes instead, in fact reduced the encapsulation efficiency. High levels of cholesterol have been reported to interfere with the close packing of lipids in the vesicles by contributing to an increase in membrane fluidity which results in an increased distribution of aqueous phase within the liposomal vesicle thereby reducing the encapsulation of the hydrophobic nevirapine [46]. The encapsulation efficiency of the liposomes was not significantly affected by the substitution of egg phospholipids with synthetic egg phosphatidyl choline probably because phosphatidyl choline is the major constituent in egg phospholipid mixture. We measured the effect of the drug-lipid ratio on liposome diameter and encapsulation efficiency. The results demonstrate that the size of the drug loaded liposomes decreased significantly from 188 ± 1.2 nm to 73 ± 7.8 nm when the drug-lipid ratio was decreased from 1:10 to 1:1 (Figure 4). A reduction in the liposome size is expected to shrink the aqueous volume of the liposome resulting in lesser encapsulation of a lipophilic drug like nevirapine [47]. Furthermore, significant increase in the amount of drug loading was observed with increasing drug-lipid ratio up to 1:5 (p < 0.05)but not beyond these ratio (Figure 4).

**Figure 5:**
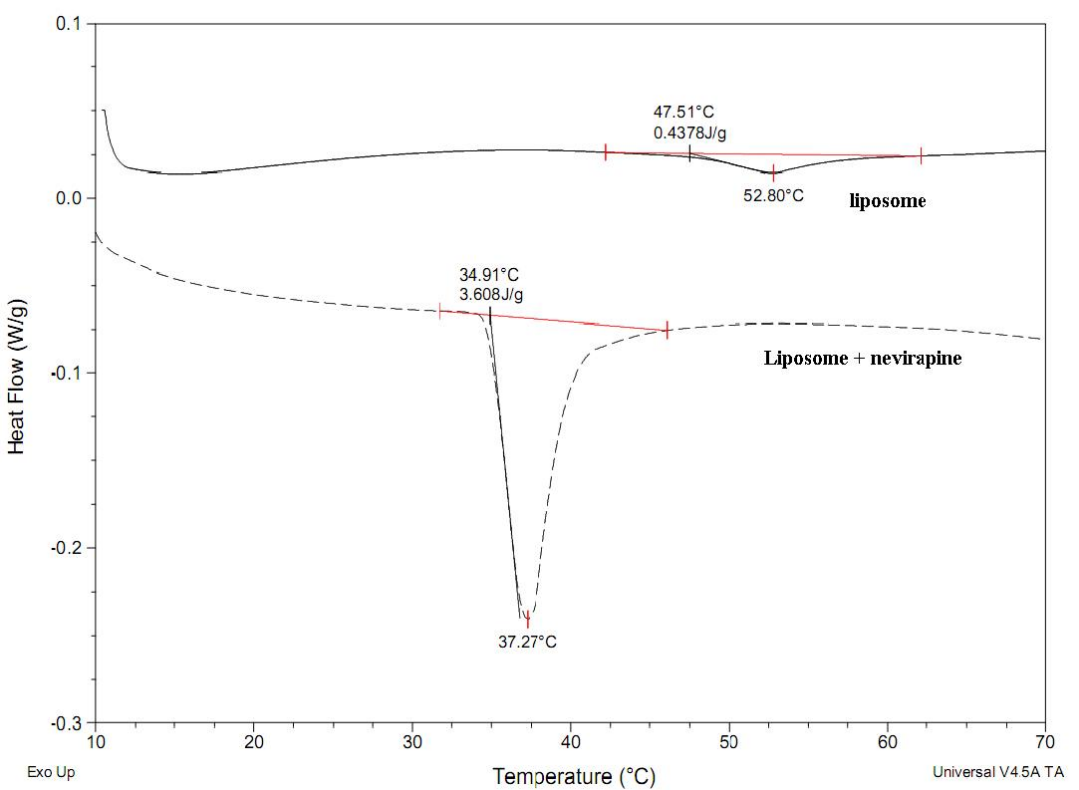
**SC thermograms of plain (filled line) and nevirapine-loaded (dashed line) liposomes**.

The encapsulation of nevirapine in the liposomes was confirmed by DSC measurements (Figure 5). The endothermic phase transition temperature of the plain liposomes and nevirapine loaded liposomes was found to be 52.80°C and 37.27°C, respectively (Figure 5). A negative shift in the transition temperature indicates a strong hydrophobic interaction between nevirapine and the phospholipids forming the liposome. The absence of flattened peaks indicates the homogeneity in the lipids forming the liposome [48].

**Figure 6:**
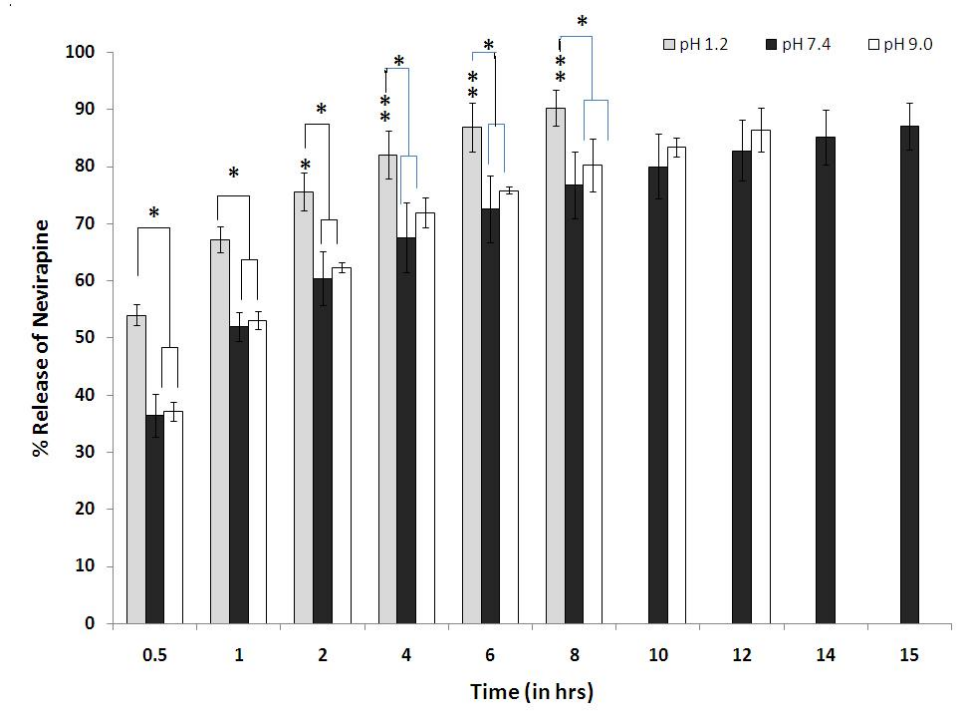
Release profile of nevirapine from liposomes at various pH containing phospholipid to cholesterol in ratio 9:1 (* < 0.05).

The drug release profile of nevirapine from the liposomes was studied at three different pH conditions (1.2, 7.4 and 9.0) and in a standard culture medium supplemented with serum. As shown in (Figure 6), during the initial half an hour, a burst release of nevirapine was observed under all the experimental conditions. Approximately, 53.98 ± 1.85%, 36.39 ± 3.68% and 37.10 ± 1.65% of the drug was released at the pH values of 1.2, 7.4 and 9.0, respectively. The magnitude of drug released at pH 1.2 was significantly higher as compared to pH 7.4 and 9.0 at all the time points (p < 0.05) (Figure 6). However, the amount of drug released at pH 7.4 and 9.0 was comparable to each other throughout the study period (p > 0.05) (Figure 6). At pH 1.2, nearly 90% of nevirapine was released within 8 hours (90.17 ± 3.13%). However, to release an equivalent amount, 15 and 12 hours will be required at pH 7.4 (87.07 ± 4.08%) and 9.0 (86.37 ± 3.86%), respectively. The faster drug release in the acidic and basic media may be attributed to the accelerated hydrolysis of the carrier [49].

**Figure 7:**
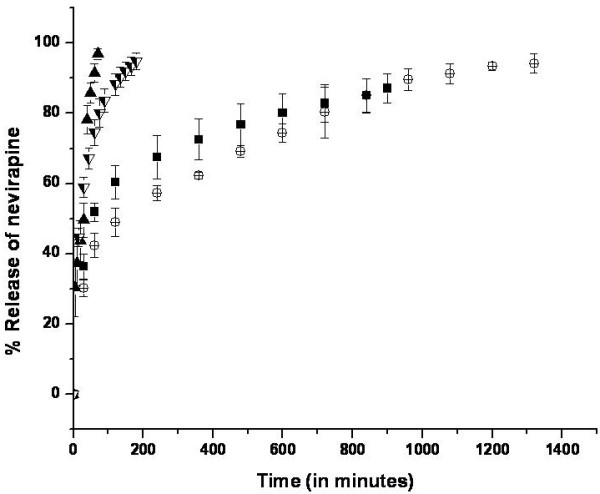
***In vitro *release profile of nevirapine in PBS (pH 7.4) release medium at various conditions**. (Black square) liposomes with phospholipid to cholesterol ratio 10:00 in PBS, (Circle with cross) Liposomes with phospholipid to cholesterol ratio 9:1 in PBS, (Black triangle) liposomes with Phospholipid to cholesterol ratio 10:00 in PBS subjected to low frequency Ultrasound and (Inverted triangle with partial shading) liposomes with phospholipid to cholesterol ratio 10:00 in DMEM cell culture medium. PBS represents the phosphate buffered saline; DMEM refers to Dulbecco's Modified Eagle's medium.

Drug release from liposomes measured *in vitro *in buffers and solutions may or may not be extrapolated to *in vivo *conditions given the highly complex composition of the physiological fluids including the presence of proteins. Ultrasound-triggered delivery systems are gaining popularity for their non-invasive nature and controlled release ability [50]. To investigate whether the synthetic liposomes demonstrate echogenic effect, we compared nevirapine release profiles from drug-loaded liposomes under different experimental conditions, in PBS or DMEM with or without low frequency ultrasound treatment (Figure 7). It was observed that 87.07 ± 4.08% of the drug was released in 900 minutes when placed in PBS as compared to 94.82 ± 2.32% in 180 minutes for DMEM and 96.86 ± 1.62% in 70 minutes while using ultrasound (Figure 7). In the first 60 minutes the percentage release of nevirapine in PBS, DMEM and ultrasound triggered release was found to be 51.92 ± 2.60%, 74.51 ± 3.74% and 91.51 ± 2.66%, respectively suggesting an initial burst release (Figure 7). The faster release in DMEM when compared to PBS may be attributed to the presence of several host derived factors including albumin protein in the cell culture medium, which could displace the phospholipids in the liposomes resulting in enhanced fluidity thereby causing a fast release of the drug [50]. Similarly most of the drug was released within 60 minutes when ultrasound was applied (Figure 7). This may be attributed to the development of highly localized pressure spots that disrupt the drug-lipid interactions and lipid-lipid interactions resulting in the observed release pattern.

**Figure 8:**
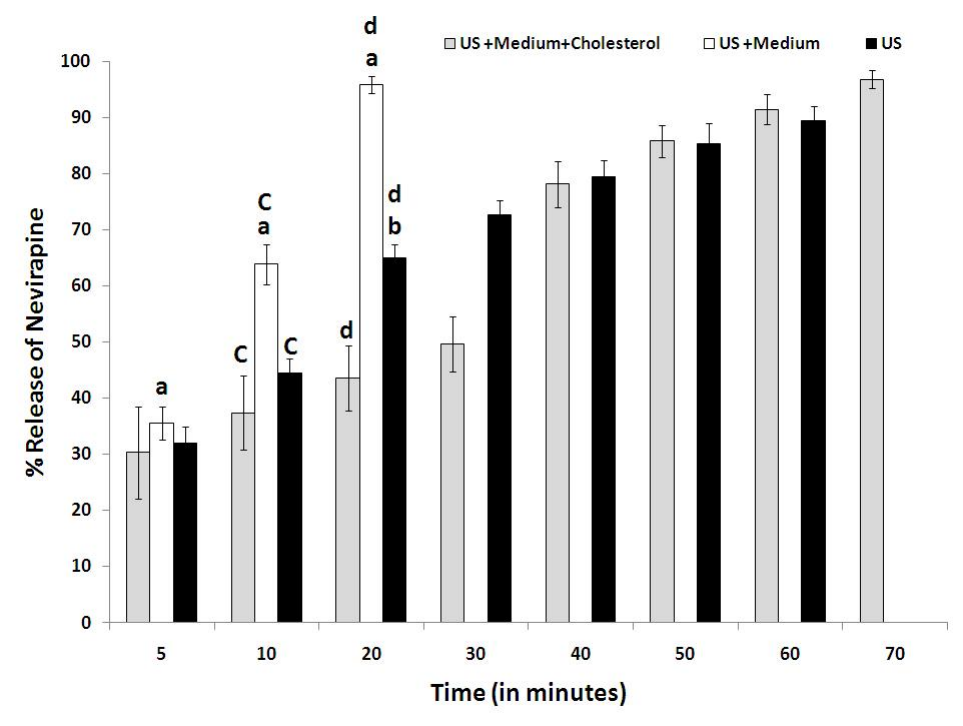
***In vitro *release profile of nevirapine in PBS (pH 7.4) subjected to ultrasound (US) under various conditions**. (Grey square) liposomes with phospholipid to cholesterol ratio 9:1 in DMEM, (white square) liposomes with phospholipid to cholesterol ratio 10:0 in DMEM, (Black square) liposomes with phospholipid to cholesterol ratio 10:0 in PBS. PBS refers the phosphate buffered saline; DMEM refers to Dulbecco's Modified Eagle's medium. Statistical data shows that a < 0.05 vs 5 minutes, b < 0.05 vs 20 minutes, c < 0.05 vs 10 minutes and d < 0.05 vs 20 minutes.

We evaluated the influence of medium and cholesterol on the ultrasound triggered release profiles from nevirapine loaded liposomes. We observed that the combined effect of medium and ultrasound accelerate the release of nevirapine from the liposomes. More than 90% of the drug was released within 20 minutes (Figure 8). The release follows a first order kinetics and the fast release may be attributed to the combined fluidization effect of proteins in the medium and ultrasound. Furthermore, when cholesterol was incorporated into the liposomes (9:1 ratio), regardless of the presence or absence of DMEM, the pattern of drug release was comparable in response to ultrasound treatment (Figures 7 and 8). This may be due to the additional rigidity conferred by cholesterol to the liposome thus preventing the displacement of phospholipids by the proteins found in the medium [51].

## Conclusions

Liposomes of uniform diameters were prepared using thin film hydration and extrusion technique and a hydrophobic non-nucleoside reverse transcriptase inhibitor, nevirapine, was successfully encapsulated in the liposomes. The best encapsulation was observed at an egg phosholipid to cholesterol ratio of 9:1 which also showed a prolonged release of nevirapine up to 1320 minutes at physiological pH. Presence of proteins in the medium and external stimuli like low frequency ultrasound was found to enhance the rate of drug release. The use of ultrasound leading to higher magnitude of drug release thus points to a potentially novel approach towards anti-retroviral therapy. Presence of cholesterol in the liposomes offers stability against fluidizing action of proteins without preventing the disruption of the liposomal architecture by ultrasound.

## Competing interests

The authors declare that they have no competing interests.

## Authors' contributions

Project was conceived and experiments designed, developed and manuscript was drafted by UMK, SS and UR. All experiments were carried out at SASTRA by LNR. All authors read and approved the final manuscript.

## Acknowledgements

The authors wish to record their gratitude to Nano Mission Council, Department of Science and Technology and Prof. T.R. Rajagopalan R&D Grant, SASTRA University, for financial support.
